# The temperament trait of environmental sensitivity is associated with connectedness to nature and affinity to animals

**DOI:** 10.1016/j.heliyon.2022.e09861

**Published:** 2022-07-05

**Authors:** Annalisa Setti, Francesca Lionetti, Rachel L. Kagari, Liam Motherway, Michael Pluess

**Affiliations:** a School of Applied Psychology, University College Cork, Ireland; b Environmental Research Institute, University College Cork, Ireland; c Department of Neuroscience, Imaging and Clinical Science, G. d’Annunzio University of Chieti-Pescara, Chieti, Italy; d Department of Biological and Experimental Psychology, Queen Mary University of London, London, UK

**Keywords:** Nature connectedness, Animal affinity, Sensory processing sensitivity, Highly sensitive person

## Abstract

Heightened sensitivity to the environment characterizes approximately 30% of the population and is associated with a higher reactivity, positive or negative, to the surrounding environment. Little attention has been devoted to study the association between this trait and the response to nature and animals, despite the potential benefits of the natural environment for highly sensitive individuals. In the present two studies (N = 241, 83% female, age M = 37.43, SD = 13.5; N = 144, 92% female, age M = 39.9, SD = 13.1) we assessed the association of sensory processing sensitivity (SPS), measured with the Highly Sensitive Person scale, with nature and animal affinity. In both studies, we found SPS to predict higher connectedness to nature. In addition, whilst there was no association between high SPS and attachment to pets in Study 1, in Study 2 SPS was predictive of a higher animal affinity, assessed in terms of stewardship and protection of animals. The present studies provide the first quantitative empirical evidence that highly sensitive individuals are more connected with nature and animals, therefore opening the possibility to explore nature based solutions to improve the quality of life in individuals scoring high in SPS.

## 1 Introduction

The importance of nature for people's well-being has become clearer to most of us in the past two years in light of the Covid-19 pandemic. People have turned to nature, and the frequency of nature contact has increased, with beneficial effects on well-being and physical health (Richardson and Hamlin, 2021). Spending at least 2 h a week in nature leads to demonstrable beneficial effects (Bratman et al., 2019), exposure to nature reduces stress (Kondo et al., 2018), and improves cognitive abilities (Berto, 2005). Walking in nature for 90 min can reduce rumination and modulates activity in the subgenual prefrontal cortex (Bratman et al., 2015). Even a brief exploration of virtual nature can benefit mood in individuals with anxiety (O'Meara et al., 2020). Given the effects on mood and rumination, exposure to nature is also beneficial for people suffering from depression (Berman et al., 2012). In the general population, nature is associated with better health and wellbeing (Hartig et al., 2014), e.g. in a study with over four thousand participants, ‘noticing nature’ was associated with increased self-reported wellbeing (Richardson and Hamlin, 2021). However, to harness the benefits of nature it is important to foster a connection with nature (Capaldi et al., 2014; Pritchard et al., 2020). Nature connectedness is the sense of being connected and part of the natural world. Those who have higher nature connectedness report to find solace in nature, to feel part of nature and to see themselves as one of the many expressions of nature, like trees and animals, and they also report being happier (Capaldi et al., 2014). Similarly, animals, particularly attachment to pets, may play an important role in combating loneliness and offering support, as shown in the recent Covid-19 lockdowns (McDonald et al., 2021). A group of individuals that might particularly benefit from nature and could be particularly connected with nature and animals are individuals scoring high in sensitivity to environmental influences (Aron et al., 2012; Lionetti et al., 2021; Pluess, 2015), that is those more sensitive to the impact of environmental stimuli as described in theories of Environmental Sensitivity (ES) (Pluess, 2015). Nonetheless no studies to date address directly whether those with high sensitivity are more connected with nature. ES is an umbrella term encompassing different conceptualizations of the relationship between the individual and the environment, with some theories emphasizing that highly sensitive individuals are more affected by environmental stressors, while other theories also focus on the evolutionary advantage of being more sensitive to the environments (see Greven et al., 2019 for a review).

A phenotypical marker of ES is Sensory Processing Sensitivity (SPS) (Aron and Aron, 1997; Greven et al., 2019), an individual trait capturing stronger reactivity to physical and emotional stimuli and the ability to process environmental stimuli in more depth (Aron and Aron, 1997; Aron et al., 2012), which has been associated with stronger responsivity to positive environments (Nocentini et al., 2018; Pluess and Boniwell, 2015) as well as higher vulnerability to mental illness when exposed to unfavorable environments (Liss et al., 2005). For example, individuals high in SPS are more at risk for depression in adulthood (Liss et al., 2008), internalizing problems (Lionetti et al., 2019) and rumination (Lionetti et al., 2021) in childhood, when the environment is less than optimal. Also, meta-analytic data provided evidence for SPS to be related to negative affect and neuroticism (Lionetti et al., 2019). This latter finding, in particular, has been proposed as potential mechanism linking sensory processing sensitivity to psychological distress (Wyller et al., 2017). Importantly, nature contact has been repetitively shown to have a positive effect on mental health issue, probably due to its positive impact in decreasing rumination strategies to cope with negative feelings (Bratman et al., 2015). Hence, it might have important implications for sensitive individuals, who may especially benefit from nature by alleviating rumination and improving mood. One qualitative study showed that being connected with nature is an important contributor to wellbeing in highly sensitive people (Black and Kern, 2020). However, to date no empirical study addressed directly the question of whether high environmental sensitivity is associated with higher nature connectedness. Given the importance of nature connectedness to foster nature benefits, and, in turn the potential of nature benefits for high SPS individuals, whether highly sensitive people are more connected with nature than lower sensitive people is a topic of interest. In particular high SPS individuals are characterized by aesthetic sensitivity (Acevedo et al., 2018; Aron et al., 2012; Lionetti et al., 2018), therefore potentially more sensitive to the beauty of nature, and they have a disposition to experience awe, a sense of admiration and ‘feeling small’ in front of nature or artifacts such as monuments (Keltner and Haidt, 2003); they are also prone to empathy, which could potentially connect highly sensitive individuals with the natural environment more than lower sensitive individuals. Interestingly, thus far, only the role of the social environment has been explored in relation to this trait (Greven et al., 2019), with no studies to date assessing specifically the association between nature connectedness and SPS.

Another important component related to the natural world is connectedness to animals. Contact with animals has been shown to be beneficial in populations other than those who are high SPS also characterized by enhanced sensitivity to environmental stimulation, e.g. Autism Spectrum Disorder (ASD) (O'Haire, 2017). While SPS differs from ASD both behaviourally and neurologically, as it is characterized by differential activation of brain regions linked to reward, empathy, self-control and depth of processing, they may share a heightened reactivity to sensory stimulation (Acevedo et al., 2018). Although specific literature on the association between SPS and animals is lacking, given their empathy, it is plausible that high SPS individuals would enjoy the company of animals and take interest in animal welfare. This is in line with the stronger emotional reactivity found in individuals scoring high in SPS (Lionetti et al., 2018), as they may benefit more from the interaction with animals, as well as perceive more deeply animals' distress and hence be more prone to protect animals. A recent study on animals' sensitivity showed that dogs also portray different levels of sensitivity and their wellbeing is enhanced when the sensitivity of the animal and the owner match (Bräm Dubé et al., 2020).

In the two current studies we investigated nature and animal connectedness in people with different levels of SPS; we hypothesised that SPS is positively associated with nature connectedness and animal affinity. Given that individuals scoring high in ES are potentially more at risk to experience internalizing symptoms, and considering the positive effects of nature interventions on the reduction of rumination, anxiety, and depression, the current study could represent an important step in identifying strategies to promote wellbeing of highly sensitive individuals. This is the first set of studies addressing this topic directly.

## 2 Study 1

### 2.1 Method

#### 2.1.1 Participants

Two hundred and forty-one participants were recruited through a University College Cork university-wide student email, advertisement on social networks, and word of mouth and via student email recruitment efforts. The sample size met the criteria of 82 according with the rule of thumb (N > 50+8m where m is the number of independent variables, in our case N > 50+ (8∗4) = 82) (Tabachnick and Fidell, 2007). The gender breakdown of the study portrayed a gender imbalance with 201 female (83.4%) and 40 male (16.6%) participants. The age range of the participants was 16–73, M = 37.43, SD = 13.5. The study received ethical approval from the UCC School of Applied Psychology Ethics Committee, which is a subcommittee of the Social Research Ethics Committee, in conformity with the 1964 Declaration of Helsinki and its later amendments. Participants provided written informed consent by ticking the consent box in the online survey.

#### 2.1.2 Materials

The online survey used previously established measures. To investigate Environmental Sensitivity, the 27-item measure (Aron and Aron, 1997) Highly Sensitive Person Scale (HSP) was used. Items include statements regarding startling easily, lower sensory thresholds and depth of processing (‘Are you easily overwhelmed by strong sensory input?/Do you seem to be aware of subtleties in your environment?/Do other people's moods affect you?) For the investigation of nature connectedness we used the 14-item Connectedness to Nature Scale (Mayer and Frantz, 2004) assessing the sense of belonging to the natural world (‘I often feel a sense of oneness with the natural world around me./I think of the natural world as a community to which I belong./I recognise and appreciate the intelligence of other living organisms.); for assessing affinity to pets, we used the 9-item Short Attachment to Pets Scale, SAPS (Marsa-Sambola et al., 2016) (‘I love pets./My pet makes me feel happy./I consider a pet to be a friend.). The scales used in this study produced acceptable levels of internal reliability. The HSP scale has Cronbach alpha of α = .918. The Connectedness to nature scale had a Cronbach alpha of α = .738 and the Short Attachment to Pets Scale had α = .937.

Participants were also asked to provide information on age, gender, nationality and years of education (school leaver/secondary school/certificate level/Bachelor's Degree/Master's Degree/PhD).

#### 2.1.3 Procedure

The survey was hosted online on the platform Qualtrics, and willing participants were presented with a link which brought them to the information sheet and consent form. Participants were required to consent to take part before entering the main body of the survey. In the main part of the survey participants were asked to provide demographic information and answered the questions on HSP, Nature Connectedness and SAPS. At the end of the survey participants were thanked for their participation and provided with the researchers contact details in case they had any questions.

#### 2.1.4 Approach to data analyses

Spearman correlations were calculated between the variables included in the study (in order to include categorical variables). In order to test the association between nature and animal affinity (dependent variables) and ES, as captured by the SPS trait, we conducted two separate multiple linear regressions with covariates age, gender, years of education, and with HSP scores as the independent variable. The assumptions for multiple linear regression were met. Statistical Package for the Social Sciences (SPSS) was utilised for descriptive statistics and regression models.

### 2.2 Results

**Figure 1:**
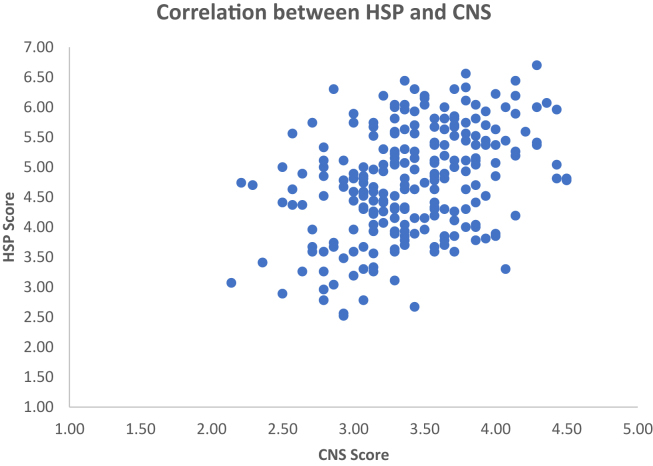
Individual scores for the Nature Connectedness Scale and the HSP scale in Study 1. Correlation between Connectedness to Nature scale (CNS) and the Highly Sensitive Person scale (HSP), in Study 1. There was a significant positive correlation with increasing HSP associated with higher connectedness to nature.

**Figure 2:**
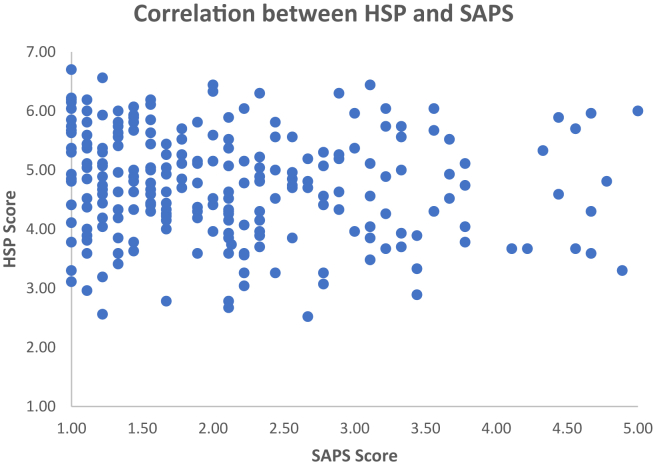
Individual scores for the SAPS and the HSP scale in Study 1. Correlation between Highly Sensitive Person Scale (HSP) and Short Attachment to Pets Scale (SAPS), in Study 1. Increasing values in the SAPS indicate *lower* attachment to pets. There is a significant negative correlation between these two variables.

The Connectedness to Nature Scale (CNS) had a mean score of M = 3.42, SD = .46 (5 point Likert Scale), the Short Attachment to Pets Scale produced a mean score of M = 2.10, SD = .96 (5 point Likert Scale). Lastly, the Highly Sensitive Person Scale which measured individual differences in ES had a mean score of M = 4.79, SD = .90 (7 point Likert Scale). The HSP score correlated positively and moderately with CNS (see Figure 1), HSP had also a significant but weak negative correlation with education and a weak and positive with gender (see Table 1). A weak and negative association was found between HSP and SAPS (higher scores indicate lower attachment) (see Figure 2). The level of education had a small but significant correlation with HSP and with CNS, while it correlated positively with SAPS, indicating that with increasing education, ES, attachment to nature and to pets diminished (see Table 1).

**Table 1:** Correlation between the variables included in Study 1.

	Age	Gender	Education	CNS Mean	SAPS Mean	HSP Mean
Age	-					
Gender	.115	-				
Education	.174∗∗	.144∗	-			
CNS Mean	.105	-.015	-.184∗∗	-		
SAPS Mean	.022	-.052	.135∗	-.339∗∗	-	
HSP Mean	0.061	.160∗	-.170∗∗	.383∗∗	-.171∗∗	-

Numbers represent the Spearman correlation coefficients. Significant correlations are indicated with an asterisk (p < 0.05∗; p < 0.01∗∗).

Two multiple linear regressions were run. The first was carried out to investigate whether Sensory Processing Sensitivity could predict participants' nature connectedness when controlling for co-variates (Table 2). The model significantly predicted nature connectedness, F (4,236) = 12.37, *p* < .001, explaining 15.9% of the variance, with HSP being positively associated to CNS, as shown in Table 2 (A). See Figure 1 for individual participants’ data.

**Table 2:** Linear regression results predicting the mean score for the Connectedness to Nature Scale (A) and Short Attachment to Pets Scale (B).

Variable Connectedness to Nature Scale (A)	B	SE B	β	t	p	CI
(Constant)	2.649	.221		11.987	.0001	2.21–3.08
Age	.004	.002	.116	1.936	.054	.000–.008
Gender	-.066	.075	-.054	-.879	.380	-.215–.082
Years of Education	-.043	.027	-.099	-1.601	.111	-.096–.01
HSP (mean)	.191	.031	.376	6.144	.0001	.130–.252
**Variable SAPS (B)**						
(Constant)	2.342	.501		4.671	.0001	1.35–3.329
Age	.001	.005	.017	.264	.792	-.008–.01
Gender	-.081	.171	-.031	-.471	.638	-.418–.257
Years of Education	.103	.061	.113	1.683	.094	-.018–.224
HSP (mean)	-.114	.071	-.108	-1.620	.107	-.254–.025

The second regression was carried out to investigate whether ES, as captured by the HSP scale, was a significant predictor of participants’ attachment to pets. The results of the regression models were not significant, F (4,235) = 1.81, *p* = .126, and the association between sensitivity and animal affinity was small and not significant (B = -.114 SE = .071 p = .107). None of the variables was a significant predictor (Table 2 B). See Figure 2 for individual data points.

### 2.3 Discussion

The first hypothesis, of a significant association between sensory processing sensitivity (HSP scale) and nature connectedness (CNS) was verified, while the second hypothesis, related to the association between SPS and connectedness with animals (SAPS) was not verified, although there was a correlation in the expected direction. We therefore set out to replicate the connectedness with nature finding and to further explore the hypothesised association with animal affinity using a different measure.

## 3 Study 2

The second study was devised to replicate Study 1 with a separate sample of participants in relation to the expected positive association between nature connectedness and ES. It was also devised to test whether the weak and non-significant association between connectedness with animals and SPS could be due to the fact that SAPS used in Study 1 captures the importance of pets in one's life, which implicitly assumes that people have a pet or are familiar with pets which may not be the case for all participants. In order to capture the connection with the animal world that may go beyond pet ownership, we adopted a scale with a broader scope. Therefore in the present study the SAPS was replaced by the Animal Attitude Scale (AAS) (Herzog et al., 2015). While the SAPS looks at affinity to animals from the perspective of interactions, especially with domestic pets, the AAS was developed to investigate dimensions such as animal welfare and humans ability to empathise with animals, which could be considered more conceptually similar to the nature connectedness scale (Mayer and Frantz, 2004).

We hypothesised that HSP would predict nature connectedness and animal affinity. The study design is the same as for Study 1.

### 3.1 Method

#### 3.1.1 Participants

One hundred and forty-four participants were recruited through university-wide email, social networks, a website on sensitivity and word of mouth. Covariates were the same as for Study 1. The gender breakdown portrayed a similar pattern to that of Study 1 in which the proportion of female participants heavily outweighed the male participation, there were 133 females (92.4%) and 11 males (7.6%). The age range of the participants was 19–83, M = 39.9, SD = 13.1. The study received ethical approval from the UCC School of Applied Psychology Ethics Committee, which is a subcommittee of the Social Research Ethics Committee. Participants provided written informed consent by ticking the consent box in the online survey.

#### 3.1.2 Material

The scales used were the Highly Sensitive Person Scale (Aron and Aron, 1997), and the Connectedness to Nature scale (Mayer and Frantz, 2004) already used in Study 1 and the Animal Attitude Scale (AAS) (Herzog et al., 2015), a five item scale assessing the attitude towards human relation with animals (‘It is morally wrong to hunt wild animals just for sport./I sometimes get upset when I see wild animals in cages at zoos.). The Cronbach alpha for the HSP scale was α = .912. The Connectedness to Nature Scale had a strong internal reliability producing a Cronbach α = .843. The AAS produced an acceptable level of internal reliability with a standardized α = .720. The covariates were the same utilised for Study 1.

#### 3.1.3 Procedure

The procedure was the same as for Study 1.

#### 3.1.4 Approach to Data Analysis

The approach to data analysis was the same as in Study 1.

### 3.2 Results

The Connectedness to Nature Scale had a mean score of M = 3.47, SD = .59, while the Animal Attitude Scale produced a mean score of M = 3.37, SD = .89. Lastly, the Highly Sensitive Person Scale which measured the Sensory Processing Sensitivity of the participants had a mean score of M = 5.10, SD = .81. HSP score correlated positively and moderately with connectedness to nature and animal affinity, see Table 3.

**Table 3:** Correlation between the variables included in Study 2.

	Age	Gender	Years Education	CNS study 2	AAS mean	HSP mean Study 2
Age	-					
Gender	.048	-				
Years Education	.051	-.008	-			
CNS study 2	.177∗	-.012	-.036	-		
AAS mean	.061	-.036	-.066	.213∗	-	
HSP mean Study 2	.013	.121	-.033	.271∗∗	.348∗∗	-

Numbers represent the Spearman correlation coefficients. Significant correlations are indicated.

**Table 4:** Linear regression results predicting the mean score for the Connectedness to Nature Scale (Study 2) (A) and the Animal Affinity Scale (AAS) (B) with an asterisk (p < .05∗; p < .01∗∗).

Variable Connectedness to Nature Scale (A)	B	SE B	β	t	p	CI
(Constant)	2.760	.486		5.675	.0001	1.80–3.72
Age	.008	.004	.168	2.067	.041	.000–.015
Gender	-.134	.181	-.060	-.740	.460	.-493-.224
Years of Education	-.012	.046	-.021	-.257	.798	-.104–.8
HSP (mean)	.193	.059	.265	3.242	.001	.075–.310
**Variable AAS (B)**						
(Constant)	2.227	0.715		3.113	.002	.813–3.641
Age	0.005	0.005	0.071	0.897	.371	-.006–.016
Gender	-0.375	0.267	-0.112	-1.406	.162	-.903–.152
Years of Education	-0.072	0.068	-0.084	-1.060	.291	-.207–.063
HSP (mean)	0.385	0.087	0.351	4.402	.0001	.212–.557

**Figure 3:**
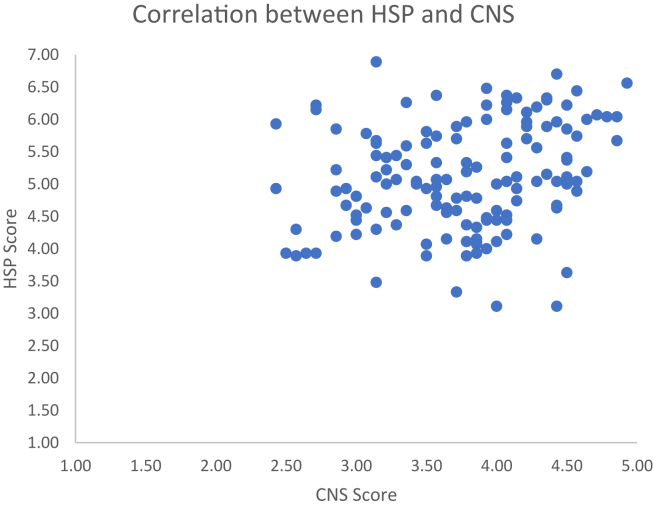
Individual scores for the Nature Connectedness Scale and the HSP scale in Study 2. Correlation between Connectedness to Nature scale (CNS) and the Highly Sensitive Person (HSP) scale, in Study 2. There was a significant positive correlation with increasing HSP associated with higher connectedness to nature.

The first regression was carried out to investigate whether the HSP scale could significantly predict participants’ nature connectedness (see Table 4A). The results of the regression indicated that the model explained a small 6.7% of the variance, and that the model was a significant predictor of nature connectedness, F (4,139) = 3.575, P = .008. Figure 3 shows the individual data points. To note, connectedness with nature also increased with age.

**Figure 4:**
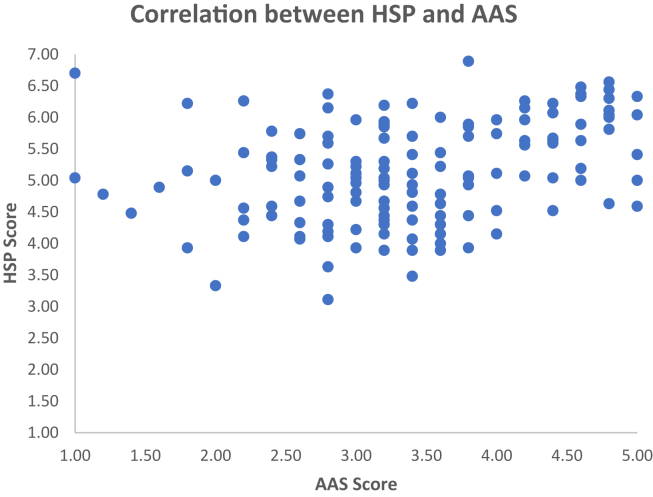
Individual scores for the AAS and the HSP scale in Study 2. Correlation between scores in the Highly Sensitive Person Scale and the Animal Affinity Scale. There was a positive correlation between these two factors, with higher HSP associated in a greater affinity to animals.

The second multiple regression was carried out to investigate whether HSP could significantly predict participants’ animal affinity (Table 4B). The results of the regression indicated that the model explained 13.5% of the variance and that the model was a significant predictor of animal affinity, F (4,139) = 5.433, P = .001. Figure 4 shows the data points of individual participants.

### 3.3 Discussion

Study 2 confirmed in a separate sample of participants that higher connectedness with nature is associated with higher sensory processing sensitivity. In addition, this study shows that when animal welfare and affinity are assessed, they are also positively associated with high sensitivity. This result confirms the importance of nature for those with higher sensory processing sensitivity, and suggests that it may include both natural environments and animals.

## 4 General discussion

A vast literature supports the benefits of nature for health and wellbeing (see e.g. Bratman et al., 2019; Hartig et al., 2014; Sumner et al.) and the importance of nature connectedness to harness these benefits (Capaldi et al., 2014). Empirical studies provided evidence that a high SPS predicts positive outcomes in response to positive stimuli (Nocentini et al., 2018; Pluess and Boniwell, 2015), but is also associated with more internalizing symptoms (Lionetti et al., 2021), especially in unfavourable social, and potentially physical, environments (Lionetti et al., 2021; Liss et al., 2005). Given these characteristics and considered the potential salutogenic effects of feeling connected with the natural world on the reduction of anxiety, depression and rumination (Barton and Rogerson, 2017), with the present studies we set to assess whether an increased SPS, as captured by the HSP scale, is predictive of an increased connectedness with nature and with animals across two independent samples. In the first study we explored whether a higher score on the HSP scale was associated with higher score on the Connectedness to Nature Scale. Results showed that feeling connected with nature characterises those who are highly sensitive more so than those who are less sensitive. We also investigated associations between SPS and attachment to animals, particularly pets. However, though the two variables showed a low but significant bivariate association, when controlling for socio-demographic variables high SPS (HSP scale) did not predict connectedness to pets anymore. Although the lack of literature on the specific topic does not allow for direct comparison of our study with others, based on findings related to the beneficial effects of animal contact in other sensory sensitive populations and on the idea that, due to their stronger emotional reaction to positive stimuli and empathy, individuals high in sensitivity might be more prone to feel connected with the natural world, including animals as pets, we expected a different outcome. In order to replicate and consolidate our finding on the association between SPS and nature connectedness and explore further the unexpected result of lack of association between SPS and attachment to pets, we conducted a second study in which we replicated the investigation of SPS and nature connectedness as in Study 1, and assessed the association between ES and animal affinity in terms of animal stewardship and welfare, without focusing specifically on love for pets. We reasoned that this scale could capture better the dimension of sensitivity to positive stimuli and empathy characterising individuals high in SPS in that connectedness with animals may not necessarily be linked to current or past pet ownership. Study 2 confirmed the expected association between nature connectedness and sensory processing sensitivity. In addition, a positive correlation between SPS and animal affinity was found, potentially capturing the dimension of connectedness with the animal world.

Both studies show that higher SPS is associated with higher connectedness with nature and, in Study 2, with animals, suggesting that interventions focused on contact with nature, for e.g. walking in nature or even just exploring nature in virtual reality, which previously proved to be effective in reducing negative affect and rumination, could be especially important for individuals high in SPS. Given the cross-sectional and correlational design of this study, this remains a suggestion to be tested in future experimental and intervention research. Two further limitations should be noted: first the sample is strongly biased towards women and towards high SPS; a more gender balanced sample and a wider spread of sensitivity scores could provide more generalisable data. While the sampling strategy targeted online outlets accessible to female and male individuals, for the vast majority female participants took part in the study. The issue of SPS being associated mostly with women is well known, although high sensitivity can characterise men as well. Whether this is a cultural issue can be established with cross-cultural studies, or by assessing SPS in population-representative studies, both of which were beyond the scope of this work. Nonetheless it should be acknowledged that a more gender-balanced sample is necessary for a more reliable generalization of the results. Second, we included a limited set of covariates in the present study, however it is of potential interest to explore the role lifestyle factors that may correlate both with nature and animal connectedness and with high SPS could modulate such association, for example cultivating mindfulness or practicing outdoor sports, as well as opportunities for nature contact. Future research could investigate where and how people with different levels of SPS engage with nature and what are the preferred activities. In is worth noting that nature connectedness is an independent construct from nature engagement and they are both predictive of outcomes (e.g. Richardson et al., 2020).

Although the preliminary nature of our finding should be acknowledged, it deserves further exploration as it has important theoretical and applied implications. First it adds to the characterization of the SPS trait, opening the possibility to investigate cognitive restoration (Kaplan, 1995; Stevenson et al., 2018) and stress recovery theory (Ulrich et al., 1991) in this population. As individual differences are becoming of particular interest in Environmental Psychology, this is a potential important avenue for further research. Second it contributes to the literature on SPS, as to date only one qualitative study explored the relationship of SPS with nature. Our study, despite its correlational design, which limits its scope, does represent a first step in understanding the role that natural environments can play in SPS. In applied settings, nature and animal contact can be a potential source of prevention and recovery from stress, and nature-based therapies could be potentially effective in such population. Implications are not only at individual level, in fact, as our cities need to cater for the diversity of the populations living in them, and considering the particular vulnerability of high SPS individuals to sensory overstimulation, knowledge about SPS and its relationship with the natural world can have implications for urban design and policy making in relation to liveability in cities (Bratman et al., 2019). Finally, with the growing urgency of the climate crisis (Watts et al., 2019), this paper suggests that individuals with high SPS, having a particular sensitivity to the welfare of nature and animals, may be more vulnerable to suffer from the destruction of nature, i.e. eco-grief and eco-anxiety (Cunsolo et al., 2020), and, conversely they may become promoters of ecological and sustainable behaviours and ambassadors for climate change in society.

## Declarations

### Author contribution statement

Setti, Annalisa: Conceived and designed the experiments; Analyzed and interpreted the data; Wrote the paper.

Lionetti Francesca: Conceived and designed the experiments; Analyzed and interpreted the data; Contributed reagents, materials, analysis tools or data.

Kagari, Rachel: Conceived and designed the experiments; Performed the experiments; Analyzed and interpreted the data.

Motherway, Liam: Analyzed and interpreted the data; Wrote the paper.

Pluess, Michael: Conceived and designed the experiments.

### Funding statement

This research did not receive any specific grant from funding agencies in the public, commercial, or not-for-profit sectors.

### Data availability statement

Data included in article/supp. material/referenced in article.

### Declaration of interest's statement

The authors declare no conflict of interest.

### Additional information

No additional information is available for this paper.

## Appendix A Supplementary data

Study1_revScoreCorr_July2021 Study1_revScoreCorr_July2021

Study2_revScoreCorr_July2021 Study2_revScoreCorr_July2021
